# Synthesis of High-Molecular-Weight Multifunctional Glycerol Polyhydroxyurethanes PHUs

**DOI:** 10.3390/molecules21091220

**Published:** 2016-09-11

**Authors:** Bassam Nohra, Laure Candy, Jean-François Blanco, Yann Raoul, Zéphirin Mouloungui

**Affiliations:** 1 Laboratoire de Chimie Agro-industrielle (LCA), Université de Toulouse, INRA, INPT, Toulouse, France; bassam@nohra.net (B.N.); laure.candy@ensiacet.fr (L.C.); 2 Laboratoire de Génie Chimique, Université de Toulouse, CNRS, INPT, UPS, Toulouse, France; jeanfrancois.blanco@ensiacet.fr; 3 Oléon SAS, Venette-BP2069-60206 Compiègne CEDEX, France; Yann.RAOUL@oleon.com

**Keywords:** polyhydroxyurethanes, glycerol carbonate acrylate, dimethyl carbonate, Aza-Michael, aminolysis, type-AB polymerization

## Abstract

Glycerol carbonate acrylate is a 5-membered cyclic carbonate synthesized from glycerol that is used as a chemical coupling agent and has proven highly suitable for use in the synthesis of multifunctional polyhydroxyurethanes (PHUs). The multifunctionality of the structure of PHUs is determined by the density of the carbon-amine groups generated by the Aza-Michael reaction and that of the urethane groups and adjacent primary and secondary hydroxyl groups generated by aminolysis. Glycerol carbonate acrylate is polymerized with polyfunctional mono-, di-, tri, and tetra-amines, by type-AB polyaddition, either in bulk or in solution, through stepwise or one-pot reaction strategies in the absence of added catalysts. These approaches result in the generation of linear, interchain, and crosslinked structures, through the polyaddition of linear and branched amines to the ethylene and cyclic carbonate sites of glycerol carbonate acrylate. The resulting collection of organic molecules gives rise to polyethylene amino ester PHUs with a high molar mass, exceeding 20,000 g·mol^−1^, with uniform dispersity.

## 1. Introduction

In 2011, Keul et al. [1] introduced the concept of using bifunctional coupling agents in the development of multifunctional polymers. Unsaturated functional cyclic carbonate monomers had long been used in the development of polar and reactive polymers [2,3]. These monomers have a 5-membered cyclic carbonate structure which contributes to their high dielectric constant, density, viscosity, and boiling point [4]. The CO_2_ sequestration at the cyclocarbonate group confers polarity and reactivity to the monomers and polymers which contain these groups. Among unsaturated functional cyclic carbonate monomers, it has been widely demonstrated that acrylate or methacrylate monomers can be easily polymerized to generate high-molar mass thermosetting polymers [2,3,5]. These polymers react with amines via their cyclic carbonate groups, thereby generating the necessary flexibility and hardness without the need to use isocyanate, which is both hazardous and toxic [2,3,5,6,7].

This sequential approach, based on the synthesis of cyclic carbonate monomers by CO_2_ fixation, generating dangling cyclic carbonate groups, followed by the generation of polar polymers with hydroxyurethane groups but without isocyanates, has undergone radical changes both in academic research and in applications for the production of organic coatings [2,3,6,7,8]. However, monomer synthesis is still based on 2,3-epoxypropyl chemistry. This molecule is obtained from petroleum [2,9,10] and the carbon footprint of synthesis therefore remains high due to the synthesis of polyacrylates and polymethacrylates, with the risk of generating non-biodegradable organic hydrocarbon polymers [11,12].

Since 1998, we have been developing procedures for preparing cyclic carbonate chemical coupling agents from glycerol, for the synthesis of multifunctional glycerol monomers, oligomers, and polymers [13,14,15,16,17,18,19]. The monomer at the heart of these chemical processes is glycerol carbonate [13,14,15,20]. This glycerol-based chemical coupling agent was identified in 2004 in France [21]. The US Department of Energy Report entitled Top Value-Added Chemicals from Biomass identified glycerol as one of 12 key renewable building-block chemicals with great promise in terms of versatility and sustainability [22]. In 2013, we identified glycerol carbonate acrylate (GCA) as a cyclic carbonate of glycerol suitable for use in the development of multifunctional polyhydroxyurethanes (PHUs) [10]. We showed that the mixing of GCA and monoalkylamines resulted in two reactions: an Aza-Michael reaction and an aminolysis reaction [23]. The Aza-Michael reaction adds an amine at the ethylene site of GCA. This condensation reaction creates carbon-nitrogen bonds. The nitrogen of the primary amine then forms new secondary and tertiary intermolecular bonds. This increases the density of the carbon-nitrogen bonds and of ethylene and carboxyester sites on GCA. During this condensation, the cyclic carbonate core of the molecule remains closed. We have shown that the condensation of the monoalkylamine begins preferentially at the ethylene site. An aminolysis reaction then occurs between the amine and cyclocarbonate sites. This reaction generates two urethane groups and two adjacent primary and secondary hydroxyl groups.

In this study, we investigated the potential of GCA for use as a cyclic carbonate based chemical coupling agent in the production of multifunctional high-molar mass PHUs. The Aza-Michael reaction generates functional linear and branched mono, di-, tri-, and tetra-amines and increases the density of nitrogen-carbon bonds. The aminolysis reaction increases the density of urethane and hydroxyl units. Linear, interchain, and crosslinked structures result from this increase in urethane unit density and from increases in the densities of adjacent primary and secondary hydroxyl groups, and ethylene and carboxyester groups, favoring the formation of multifunctional PHUs.

## 2. Results

### 2.1. Reaction Schemes for the Synthesis of Glycerol Amino Ethylene Ester PHUs

**Figure 1:**
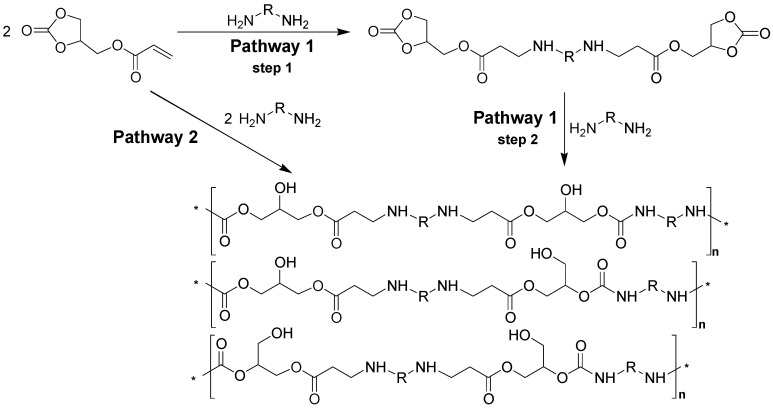
Synthesis of a glycerol amino ethylene ester PHUs (R = C_2_H_4_, C_6_H_12_, C_6_H_15_N_2_).

Stepwise type-AB polymerization to provide a linear glycerol amino ethylene ester PHUs can be achieved by two strategies (Figure 1): a stepwise reaction strategy and a one-pot reaction strategy. In the stepwise strategy (steps 1 and 2), the first step is the formation of a bicyclic carbonate compound through the stoichiometric Aza-Michael addition of diamine groups to the glycerol carbonate acrylate (step 1). The addition of a second mole of diamine to the cyclic groups leads to the production of glycerol amino ethylene esters PHUs (step 2). The second strategy (pathway 2), based on a one-pot reaction, involves the addition of larger quantities of diamine than of GCA, and leads to the formation of glycerol amino ethylene ester PHUs.

We initially investigated the effects of certain experimental parameters, by studying the self-reaction of GCA at a temperature of 90 °C under mechanical stirring. No degradation or polymerization of GCA was observed.

### 2.2. Stepwise Strategy for the Synthesis of Glycerol Amino Ethylene Ester PHUs

We studied the Aza-Michael addition of a diamine (HMDA) to GCA double bond (pathway 1). We used two moles of GCA per mole of diamine and we obtained a highly viscous solution of bicyclic carbonates with a complete consumption of acrylate functions. Given the reactivity of GCA, this first step inevitably led to the formation of small amounts of polymer in the reaction medium, also increasing its viscosity. Polyaddition, to generate the PHU polymer, was then initiated by adding a second mole of diamine per mole of bicyclic carbonate. This reaction had a very low conversion rate (about 32%) and led to the production of a hydroxyurethane oligomer. The poor miscibility of the bicyclic carbonates/diamine (or triamine) system determined the conversion rate of the reactants. The stepwise strategy, even if it is selective, is responsible for a lack of reactivity for the GCA bifunctional coupling agent.

### 2.3. One-Pot Strategy for the Synthesis of Glycerol Amino Ethylene Ester PHUs

Glycerol amino ethylene ester PHUs were preferentially synthesized via pathway 2. Several preliminary studies by polyaddition were performed without solvent and at a temperature of 90 °C. We then varied several parameters: the nature of the amine, the reaction temperature, the molar ratio of GCA to amine, and reaction in the presence or absence of solvent. All these parameters would be expected to influence the characteristics of the polymers formed. We will focus here, in particular, on the impact of these parameters on the molar mass of the polymer and its dispersity index.

#### 2.3.1. Effect of the Nature of the Amine

We reacted two moles of GCA with two moles of diamine (or three moles of GCA with two moles of triamine), without solvent, at a temperature of 90 °C. By using different types of amines (chain length, functional groups, order), we were able to obtain various glycerol amino ethylene ester PHUs (Table 1). We noted clear differences in number-average and mass-average molar mass between the various polymers synthesized. The values obtained were markedly higher than published data. The conversion rate was affected by the nature of the amine used. The glycerol carbonate conversion rate was below 100% in all cases, indicating that cyclic carbonate residues remained present in the medium. Proton NMR and infrared spectroscopy also demonstrated the presence of these residues in the mixture. However, these studies also showed that all the double bonds of GCA were consumed.

The use of a secondary diamine precluded PHUs formation, resulting in a conversion rate of 0%. Indeed, characterization of the mixture showed a complete absence of cyclic carbonate consumption, consistent with the low reactivity, in Aza-Michael reactions without solvent, of secondary amines not functionalized by cyclic carbonate moieties. Moreover, the steric hindrance and reactivity of the amine may be important parameters in the polyaddition of diamines to GCA [3,24]. The reaction of GCA with a secondary amine resulted in the formation of 100% bicyclic carbonates when dimethyl carbonate (DMC) was used as the solvent [23].

These syntheses generated linear glycerol amino ethylene ester PHUs from GCA and a linear diamine. Molar mass decreased with the length of the aliphatic chain. Branched linear polymers were also obtained in reactions using an amine with primary and secondary amine functions (trial 4). Finally, glycerol aminoester PHUs in the form of a star (or network) were obtained with high-functionality amines, such as triamines (trial 5).

#### 2.3.2. Effect of the Temperature

We performed the type-AB polyaddition reaction at three different temperatures: 60, 90, and 120 °C. At 60 °C, the conversion rate was only 40% with HMDA (Table 2). The resulting polymer had a low mean number-average molar mass, at 8500 g·mol^−1^. Infrared spectroscopy and proton NMR studies showed that residual double bonds were present. The acrylate groups of the GCA were therefore incompletely consumed. Increasing the reaction temperature to 90 °C increased the conversion rate to 75% and resulted in the generation of a glycerol amino ethylene ester PHUs with a mean molar mass of 23,300 g·mol^−1^. Increasing the reaction temperature to 120 °C had little effect on the conversion rate and the polymer obtained had a mean molar mass of 25,400 g·mol^−1^.

**Figure 2:**
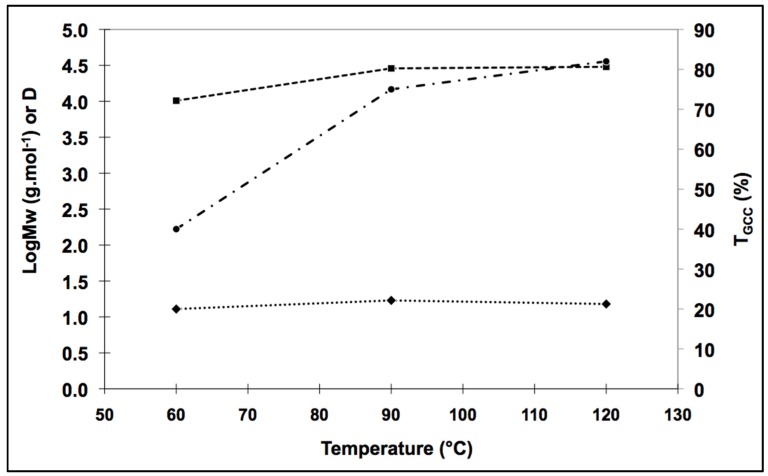
One-pot strategy; impact of the temperature on molar mass, dispersity index, and conversion rate for HMDA addition on GCA; (■) Log Mw, (♦) D, (●) T_GCC_.

Conversion rate and molar mass followed similar patterns between 60 °C and 120 °C (Figure 2). However, the dispersity index (D) drifted away from one between 60 °C and 90 °C. Synthesis at 120 °C led to the production of a molecule with a molar mass similar to that at 90 °C, but with a better D. These features suggest that a more uniform polymer was produced, consistent with the D obtained by anionic polymerization pathways [25].

Benyahya et al. [26] obtained a polymer with a number-average molar mass of 20,000 g·mol^−1^, but with a less favorable D of 2.5, in a reaction using a linear diamine as dodecadecyldiamine.

We have shown that the Aza-Michael reaction is the fastest step in our sequential polymerization approach. The concentration of the amine would, therefore, be expected to have only a small effect on reaction kinetics. We thus consider the reaction to be a pseudo-order 1 reaction, rather than a second-order reaction [27]. The kinetics of the reaction depends principally on the concentration of GCA. The rate of the reaction can thus be written:

(1) $$v=kapp\times C_{GCA}^{t}$$

After integration, the change in GCA concentration can be expressed as follows:

(2) $$C_{GCA}^{t}/C_{GCA}^{t0}=e^{-(k_{app}\times t)}$$

Taking the conversion rate into account, after 2 h of reaction, the energy of activation for this kinetic system can be defined using the Arrhenius expression, as follows:

(3) $$\text{ln}\;kapp=\text{ln}\;A-(\frac{Ea}{R})\times \frac{1}{T}$$

where *R* is the perfect gas constant, *T* is the temperature in K, and *A* is the frequency factor.

Based on measured conversion rates (Table 2), for reactions at 60 °C, 90 °C, and 120 °C, we obtain an activation energy of 22 kJ·mol^−1^. This value is consistent with published results. Tomita et al. [28] showed that the activation energy for a five-membered bicyclic carbonate was 24.9 kJ·mol^−1^.

#### 2.3.3. Effect of the Solvent

We chose to work with dimethyl carbonate (DMC), which has good solvation and environmental acceptance properties [4,29]. DMC is a polar non cyclic carbonate solvent with interesting characteristics. It can thin our viscous media by breaking intermolecular and intramolecular hydrogen bonds of intermediates oligomers and final polymers thanks to his high dielectric constant (ε = 3.087), low density (1.0636 at 25 °C), and low viscosity (0.589 cP at 25 °C). Its low boiling point of 90.5 °C at 760 mmHg is an advantage being easy recovered in substantially pure conditions after being processed. 

The use of DMC increased the conversion rate from 75% to 100%, regardless of the diamine used (Table 3). This increase in conversion rate was accompanied by an increase in the mean number-average molar mass and a slight decrease of the D. The uniformity of the polymer thus appeared to increase with conversion rate.

The use of DMC results in a particularly high concentration of reaction centers and, thus, to high conversion rates. It also helps to control the viscosity of the mixture and to regulate the reactional thermal phenomena. The resulting polymers displayed elasticity if generated with the branched polyamine TETA.

**Figure 3:**
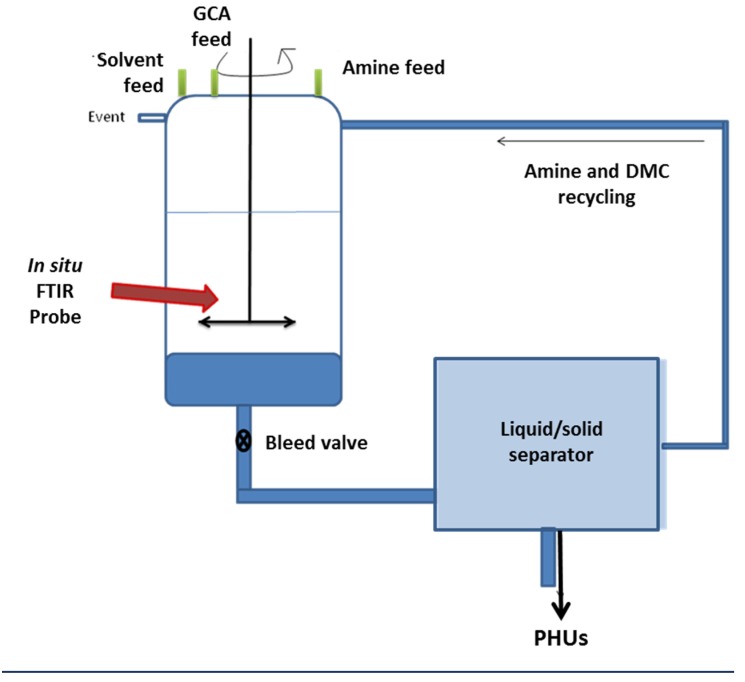
Batch process scheme for the synthesis of high-molecular-weight glycerol amino ethylene ester PHUs.

Both GCA and diamines are soluble in DMC. The medium gradually becomes heterogeneous as the polymer forms. The polymer precipitates and the DMC could therefore, be recovered by filtration at the end of the reaction. It is, therefore, possible to recycle both the solvent and the residual amine. This synthesis is sufficiently robust to be carried out in a stirred batch reactor, and it could also be scaled up. It would also be possible to transfer the process from a stirred batch reactor to a clean continuous process in which the reaction and separation sequences are coupled to DMC recycling (Figure 3).

#### 2.3.4. Effect of the GCA/Amine Molar Ratio

The effect of the GCA/amine molar ratio was evaluated for polymerization reactions in the presence and absence of DMC (Table 4). Without DMC, increasing the amount of amine (trials 2 and 11) resulted in a small increase in molar mass and a slight increase in conversion rate. This is probably due to the limited miscibility of the diamine in the medium. The medium becomes increasingly viscous towards the end of the reaction. These rheological properties were used by Benyahya et al. [26] who studied the optimization of PHU synthesis, using a dynamic rheometer to determine the best operating conditions as a function of changes in the rheological characteristics of PHU synthesis media.

If there are too few amine groups (trial 12), with a GCA/amine molar ratio of 2/1, polymerization leads to the production of low-molar mass glycerol amino ethylene ester PHUs with a low rate of conversion. Typically, 80% conversion of GCA double bonds by the Aza-Michael addition of diamine to alkene sites is observed, resulting in the generation of bicyclic carbonates. These molecules then react with the diamine, with a conversion rate of 20%. A deficiency of diamine in the medium and the very different reactivity of the two GCA groups affect the conversion rate and the characteristics of the polymer. In the high-temperature reaction conditions used, the reaction medium is a viscous liquid. This liquid forms a rigid transparent gel on cooling.

Combining the DMC effect with a stepwise addition of the diamine led to a complete reaction of the GCA alkene groups and a more than 90% conversion rate for bicyclic carbonates. It consisted in restoring the amount of amine present to equilibrium levels to obtain higher conversion rates.

The aliphatic diamine contains two primary amine sites and two secondary amine sites, all able to react in Aza-Michael reactions [23]. We tried to react each of these sites with GCA to generate additional cyclic moieties, thereby making it possible to increase the molar mass of the polymer. The addition of 4 moles of GCA per mole of amine lead to the formation of 4 cyclic carbonate groups as the GCA alkene group conversion was complete. The consecutive addition of 2 moles of amine lead to conversion rates of 90% for cyclic carbonates, corresponding to the opening of four cyclic carbonate groups. This strategy was also successfully applied to TAEA which has three primary amine groups.

### 2.4. Characterization of Glycerol Amino Ethylene Ester PHUs

**Figure 4:**
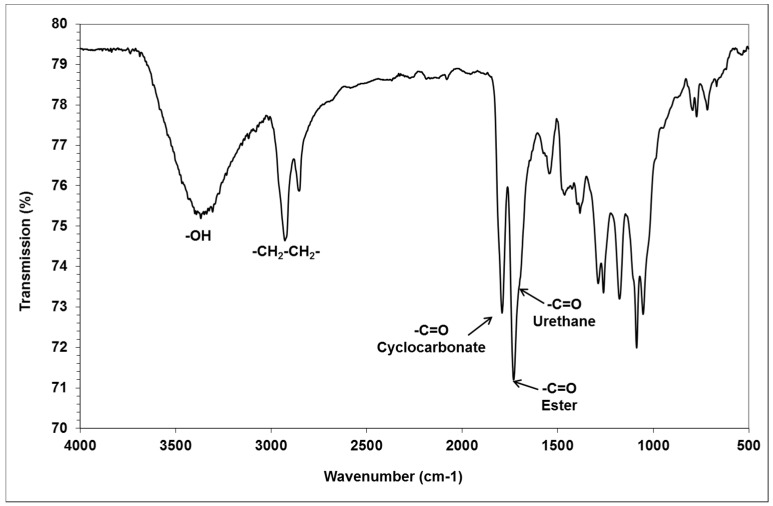
Infrared spectrum of a glycerol amino ethylene ester PHU gel (trial 12).

**Figure 5:**
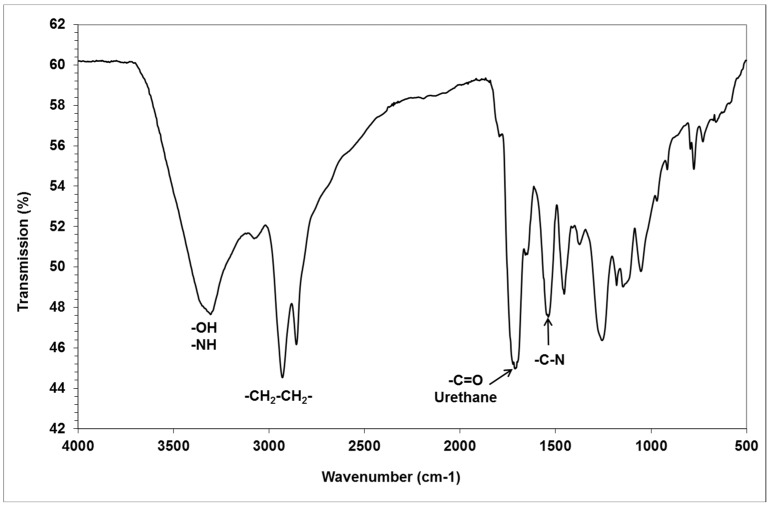
Infrared spectrum of a glycerol amino ethylene ester PHU resin (trial 9).

We characterized the glycerol amino ethylene ester PHUs, by determining the quantitative composition of the mixtures obtained. The products were analyzed by infrared spectroscopy to detect ester functions (1737 cm^−1^) and urethane functions (1705 cm^−1^). The FTIR spectrum of a low-molar mass glycerol amino ethylene ester PHUs, with incomplete consumption of the bicyclic carbonates (polymer in gel form, trial 12) is shown in Figure 4. The alkene band at 1640 cm^−1^ disappeared after the Aza-Michael addition of diamine to GCA, and the cyclic carbonate band at 1795 cm^−1^ disappeared after the complete opening of the GCA molecule by the addition of multiple diamine or triamine molecules to form glycerol amino ethylene ester PHUs (Figure 5).

**Figure 6:**
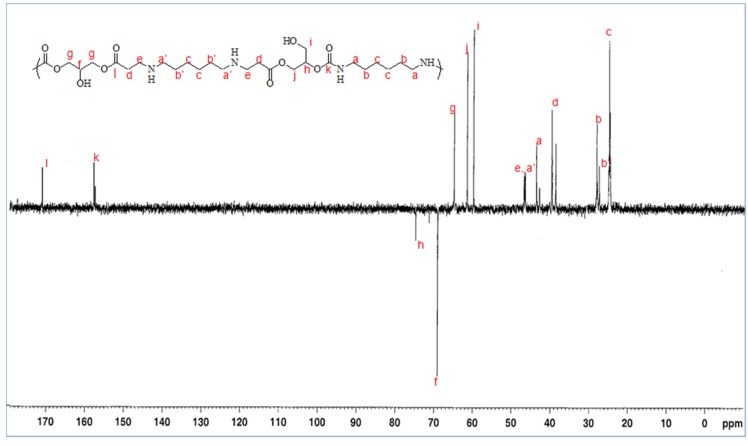
Representation and nomenclature for a glycerol amino ethylene ester PHUs for ^13^C-NMR (dept135) in D_2_O.

The attribution of the ^13^C-NMR signals is based on data obtained for glycerol amino ethylene ester PHUs with cyclic carbonate conversion rates close to 100% (Figure 6). The characteristic bands can be attributed to the carbons of the ester at 173 ppm and those of urethane at 158.94 and 158.57 ppm. The lack of resonance at 156–157 ppm confirms that the cyclic carbonate groups had opened completely to form glycerol amino ethylene ester PHUs. Furthermore, the alkyl chain carbons located at polymer chain centers (obtained by adding diamine to GCA) were found not to be identical to those at the ends of the alkyl chain (after the opening of cyclic carbonates by a diamine or triamine). Carbon a′ displays greater deshielding than carbon a.

**Figure 7:**
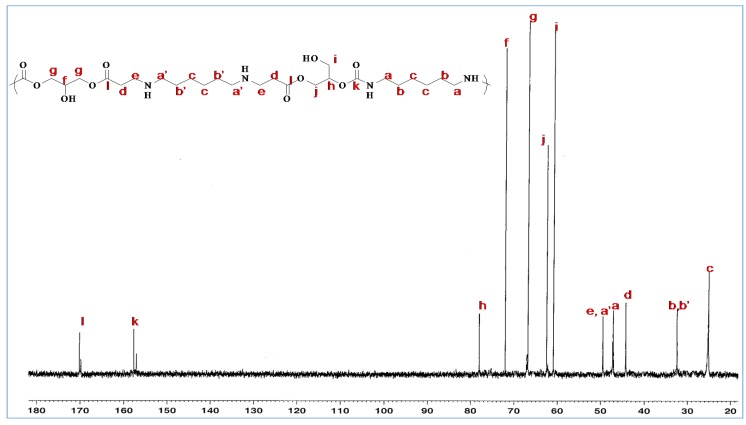
zgig ^13^C-NMR spectrum for a glycerol amino ester ethylene PHUs in D_2_O.

By integrating carbon zgig ^13^C-NMR signals (Figure 7), it is possible to calculate the OH I area/OH II area ratio of the glycerol amino ethylene ester PHU formed [30]. We calculated this ratio by comparing the integrals for characteristic groups. We integrated carbon NMR signals during zgig acquisition and obtained a 25/75 ratio perfectly consistent with published data [31].

## 3. Discussion

Glycerol carbonate acrylate is a bifunctional coupling agent that allows moving from CHO to CHON structures by reacting both the ethylene and cyclic carbonates sites of GCA by Michael Addition and condensation reaction with amines, respectively. The CHON structures combine ester, secondary and/or tertiary amine, primary and secondary hydroxyls, and urethane functions spaced with disubstituted ethylene units. These original PHUs could only be obtained by the polymerization of the specific glycerilic cyclic carbonate GCA.

We have developed two different approaches for obtaining high-molar mass glycerol amino ethylene ester PHUs from GCA by a stepwise reaction strategy and a one-pot reaction strategy, without any catalysts. The reaction can be monitored by FTIR spectroscopy to follow the conversion of alkene and cyclic carbonate groups and the formation of urethane and hydroxyl groups. The ratio of primary to secondary hydroxyl groups in the polymer can be determined by ^13^C-NMR.

With the stepwise strategy, we have demonstrated that the step 1 (Michael reaction) produces cyclic carbonates structures spaced with secondary or tertiary amines in their aliphatic chain. Due to the high reactivity of amines for the alkene sites of the acrylate, the Aza-Michael addition of amines to GCA results in the synthesis of glycerol amino ethylene ester cyclic carbonates. These bicyclic carbonates molecules are weakly reactive when adding additional amines [23]. The rate constant for Aza-Michael addition is well above that for the opening of the cyclic carbonate ring. The presence of Van der Walls bonds of N-H type competes to create in situ structured networks and are responsible for the increase in viscosity in the reactional medium. The networks are so cohesive that the consecutive insertion of amines on the cyclic carbonates moieties is limited leading to a weak opening of the cycles; the limited formation of C, H, O, N structures; and the production of low molar mass PHUs.

The one-pot strategy favors the almost simultaneous occurrence of Michael addition and condensation to produce high molar mass CHON structures. The differences in the molar masses were highlighted by the study of experimental parameters. We initially studied the bulk type AB polymerization reaction in experiments in which we varied the nature of the amine. The type of amine and the GCA/amine ratio affected the average molar mass and morphology of the polymer generated. Temperature also affected the polymerization reaction and the rate of conversion for cyclic carbonates. This conversion rate increased substantially between 60 °C and 90 °C, with no further change at 120 °C. The increasing viscosity of the mixture at higher temperatures limited reactivity.

The polycondensation is favored by varying the GCA/Amine ratio and by adding DMC as solvent. The addition of DMC resulted in polymers with a higher molar mass and a good dispersity index. This last point also opened perspectives for using DMC to allow improving the stepwise strategy. DMC acts as a diluent co-component likely to break the Van der Walls bonds and to activate the reactivity of the amine sites and their condensation with cyclic carbonates sites. Moreover, a chemical synergistic effect activating the reactive species in the medium was developed by the joint presence of DMC and intermediate glycerilic cyclic carbonates generated in situ. These bicyclic carbonate intermediates have useful polar, conductive, and activating properties. They promote autocatalytic reactions through the in situ activation of amine and cyclic carbonate sites, facilitating in particular the aminolysis in the absence of added catalysts. These activating properties are the driving force behind this new AB-type polymerization pathway for the generation of glycerol amino ethylene ester PHUs.

Our method of choice was the one-pot reaction with DMC as compatibilizer as it presented the advantages of being a clean, inexpensive, and efficient process. All of the GCA and amine atoms enter the PHUs atomic composition promoting the atom economy and an environmental efficiency [32]. No catalyst was used and no residual byproducts or co-reagents were detected in the final media. DMC also presents the advantage of being recovered and recyclable at the end of the reaction, especially since it is not contaminated with catalysts.

With the one-pot strategy, high molar masses ranging from 25,000 to 55,000 g·mol^−1^ were obtained with equivalent dispersity indexes, representative of identical hydrocarbon skeletons, typical of the –CH_2_-CH_2_– ethylenic spacer. Our synthesis pathways generated polymers in the form of gels, foams, and resins. These rigid, flexible, or elastic products have many possible applications. This type-AB polymerization can be used to generate oligoprecursors for bulk, solution, or photochemical polymerization. Various non-toxic biodegradable reactive groups are available for the production of tailor-made copolymers with specific properties for particular applications compatible with the environment.

## 4. Experimental Section

### 4.1. Materials

Glycerol carbonate (Huntsman, Port Neches, TX, USA, 99.5%), acryloyl chloride (Sigma Aldrich, Saint Quentin Fallavier, France, 97%), Ethylenediamine (EDA, Sigma Aldrich, 99%), hexamethylenediamine (HMDA, Sigma Aldrich, 98%), *N*,*N*′-diethylethylenediamine (DEEDA, Sigma Aldrich, 99%), triethylenetetramine (TETA, Sigma Aldrich, >97%), tris (2-aminoethyl)amine (TAEA, Sigma Aldrich, 96%), Dimethyl carbonate (DMC, Sigma Aldrich, 97%), were used as received.

### 4.2. Synthesis

#### 4.2.1. Synthesis of Glycerol Carbonate Acrylate (GCA)

GCA was synthesized from glycerol carbonate and acryloyl chloride and characterized as previously described [22].

#### 4.2.2. Two-Step Synthesis of Glycerol Amino Ethylene Ester PHUs

Step 1: 0.116 mole (20 g) of GCA was placed in a 100 mL reactor equipped with a 500 rpm mechanical stirring and heated to 90 °C. 0.058 mole of amine (chosen between EDA, HMDA, DEEDA, TETA, TAEA) was slowly added. The reaction was carried out for 1 h at 90 °C and the progress of the reaction was followed by FTIR. 

Step 2: 0.058 mole of amine was added, under stirring, to the preceding reaction medium and the reaction was continued for 2 h at the same temperature.

#### 4.2.3. One-Pot Synthesis of Glycerol Amino Ethylene Ester PHUs

0.116 mole (20 g) of GCA was placed in a 100 mL reactor equipped with a 500 rpm mechanical stirring and heated to 90 °C. 0.116 mole of amine (chosen between EDA, HMDA, DEEDA, TETA, TAEA) was slowly added. For reactions using solvent, 40 mL of DMC were added to GCA before heating. The reaction was carried out for 2 h at 90 °C and the progress of the reaction was followed by FTIR.

### 4.3. Characterization

#### 4.3.1. Fourier Transform Infrared Spectroscopy

Fourier transform infrared (FTIR) spectra were achieved by transmission on a PerkinElmer Spectrum 65 spectrometer (PerkinElmer SAS, Courtaboeuf, France).

#### 4.3.2. Nuclear Magnetic Resonance (NMR) Spectroscopy

^1^H and ^13^C nuclear magnetic resonance (NMR) spectra were achieved on a Bruker Advance^®^ 300 MHz instrument (Bruker Biospin S.A.S., Wissembourg, France) using tetramethylsilane (TMS) as an internal standard and equipped with QNP probe (^1^H, ^19^F, ^31^P, ^13^C). The proportions of secondary and primary OH groups were determined at 293.2 K, with zgig ^13^C pulse program. The delay time D1 is greater than or equal to 30 s in order to permit the full relaxation of all nuclei concerned in the assay.

#### 4.3.3. Size Exclusion Chromatography (SEC)

SEC was used to determine molecular masses and dispersity indexes of the polymer samples. Analyses were achieved on a system equipped with a P580 Pump (Dionex, Voisins-le-Bretonneux, France), an ASI-100 Automated sample Injector (Dionex, France), and a 350 RI detector (Varian, Les Ullis, France). Two PL Aquagel-OH30 (8 µm × 300 mm × 7.5 mm) columns in series were used at a 0.8 mL·min^−1^ flow rate with water as eluent 30 °C. The molar masses were calculated with respect to polysaccharide standards (Polymer Laboratories Mp = 180 g·mol^−1^ to Mp = 3.8 × 10^5^ g·mol^−1^).

## Figures and Tables

**Table 1:** One-pot strategy; Influence of the amine on the synthesis of glycerol amino ethylene ester PHUs. T = 90 °C, duration = 2 h.

Trial	Amine	M_n_^a^	M_w_^b^	D ^c^	Aspect ^d^	T_GCC_ (%) ^e^
1	EDA	35,600	52,400	1.43	Foam	69
2	HMDA	23,300	28,700	1.23	Rigid resin	75
3	DEEDA	-	-	-	No polymerization	0
4	TETA	32,800	44,000	1.34	Rigid resin	79
5	TAEA	42,200	53,400	1.26	Solid granules	83

^a^ Number-average molar mass (M_n_) determined by SEC; ^b^ Mass-average molecular mass (M_w_) determined by SEC; ^c^ Dispersity index (M_w_/M_n_); ^d^ Visual appearance of the polymers obtained; ^e^ Conversion rate (%) of cyclic carbonates determined by ^1^H-NMR.

**Table 2:** One-pot strategy; influence of the temperature on the synthesis of glycerol amino ethylene ester PHUs. Duration = 2 h.

Trial	Amine	T (°C)	M_n_^a^	M_w_^b^	D ^c^	T_GCC_ (%) ^d^
6	HMDA	60	8500	10,200	1.11	40
7	HMDA	90	23,300	28,700	1.23	75
8	HMDA	120	25,400	30,200	1.18	82

^a^ Number-average molar mass (M_n_) determined by SEC; ^b^ Mass-average molar mass (M_w_) determined by SEC; ^c^ Dispersity index (M_w_/M_n_); ^d^ Conversion rate (%) of cyclic carbonates determined by ^1^H-NMR.

**Table 3:** One-pot strategy; influence of DMC solvent on the synthesis of amino ethylene ester PHUs. T = 90 °C, duration = 2 h.

Trial	Amine	Solvent	M_n_^a^	M_w_^b^	D ^c^	Aspect ^d^	T_GCC_ (%) ^e^
2	HMDA	/	23,300	28,700	1.23	Rigid resin	75
4	TETA	/	32,800	44,000	1.34	Rigid resin	79
9	HMDA	DMC	27,300	31,400	1.15	Rigid resin	95
10	TETA	DMC	42,800	54,000	1.26	Elastic	96

^a^ Number-average molar mass (M_n_) determined by SEC; ^b^ Mass-average molar mass (M_w_) determined by SEC; ^c^ Dispersity index (M_w_/M_n_); ^d^ Visual appearance of the polymers obtained; ^e^ Conversion rate (%) of cyclic carbonates determined by ^1^H-NMR.

**Table 4:** Influence of the GCA/amine molar ratio on the synthesis of amino ethylene ester PHUs. T = 90 °C, duration = 2 h.

Trial	GCA/Amine ^a^	Amine	Solvent	M_n_^b^	M_w_^c^	D ^d^	Aspect ^e^	T_GCC_ (%) ^f^	T_GCA_ (%) ^g^
2	1/2	HMDA	/	23,300	28,700	1.23	Rigid resin	75	100
11	1/3	HMDA	/	25,200	29,000	1.15	Rigid resin	79	100
12	2/1	HMDA	DMC	5600	7000	1.23	Rigid gel	20	80

^a^ GCA/amine ratio of the reagents; ^b^ Number-average molar mass (M_n_) determined by SEC; ^c^ Mass-average molar mass (M_w_) determined by SEC; ^d^ Dispersity index (M_w_/M_n_); ^e^ Visual appearance of the polymers obtained; ^f^ Conversion rate (%) for cyclic carbonates determined by ^1^H-NMR; ^g^ Conversion rate (%) for ethylenic group of GCA determined by ^1^H-NMR.
